# Loss of function variants in the primate-specific gene *ZNF808* cause neonatal, transient and adult-onset diabetes

**DOI:** 10.1016/j.ebiom.2025.106113

**Published:** 2026-01-06

**Authors:** James Russ-Silsby, Kevin Colclough, Matthew B. Johnson, Matthew N. Wakeling, Nick D.L. Owens, Shenali A. Amaratunga, Sarah E. Flanagan, Kashyap A. Patel, Andrew T. Hattersley, Elisa De Franco, ZNF808 clinical consortium, Mohamed Abdullah, Hessa Alkandari, Zehra Aycan, Semra Çetinkaya, Nancy Elbarbary, Radha Ghildiyal, Susana Gonzalez, Shaun Gorman, Samar Hassan, Savita Khadse, Jan Lebl, Jaida Manzoor, Nikhil Shah, Tara Hussein Tayeb, the Human-Specific Pancreatic Development Consortium, Alaa Al Assi, Arya Anil, Diego Balboa, Urvashi Chitnavis, Juliette Davis, Doga Eskier, Michael Imbeault, Santiago Morell, Sachin Muralidharan, Timo Otonkoski, Jonna Saarimäki-Vire

**Affiliations:** a Department of Clinical and Biomedical Sciences, University of Exeter, Exeter, United Kingdom; b Exeter Genomics Laboratory, Royal Devon University Healthcare NHS Foundation Trust, Exeter, United Kingdom; c Department of Pediatrics, 2nd Faculty of Medicine, Charles University and Motol University Hospital, Prague, Czechia

**Keywords:** *ZNF808*, Pancreatic agenesis, Neonatal diabetes, Variable phenotype

## Abstract

**Background:**

Biallelic loss-of-function *ZNF808* variants were recently identified as a cause of pancreatic agenesis characterised by insulin-treated permanent neonatal diabetes (PNDM), low birthweight and exocrine pancreatic insufficiency.

**Methods:**

We investigated the phenotypic diversity caused by biallelic loss-of-function *ZNF808* variants by screening a cohort of 4699 individuals with genetically undiagnosed monogenic diabetes: 335 with neonatal diabetes (NDM, diagnosed <6 months), 194 with infancy-onset diabetes (diagnosed 6–12 months) and 4170 diagnosed with diabetes between 1 and 60 years of age.

**Findings:**

Through a combination of genome and targeted next-generation-sequencing, we identified 17 previously unreported individuals with biallelic loss-of-function *ZNF808* variants, bringing the total number of cases identified in the Exeter cohort to 31 when combined with previously described cases. 30/31 individuals were born to related parents. Clinically, 19 had PNDM, whilst the remaining 12 had other diabetes phenotypes: 5 with infancy-onset diabetes, 4 with transient diabetes and 3 with diabetes diagnosed aged 10, 14 and 23 years. Individuals with *ZNF808*-diabetes did not always require insulin treatment, with sulphonylurea treatment reported in 3 individuals. Exocrine pancreatic function was not consistently affected across the cohort, with no clinical features of exocrine insufficiency reported in 17 individuals and normal exocrine function biochemically confirmed in one further individual.

**Interpretation:**

Biallelic loss of *ZNF808* results in a variable pancreatic phenotype ranging from pancreatic agenesis to adult-onset diabetes without exocrine insufficiency. *ZNF808* gene testing should be considered in individuals with diabetes diagnosed after the neonatal period, especially if born to related parents.

**Funding:**

10.13039/100010269Wellcome Trust, 10.13039/501100000361Diabetes UK.

**Research in context**

**Evidence before this study**

A search of the terms “*ZNF808*” and “Diabetes” on PubMed resulted in only 4 research article entries: the article identifying *ZNF808* variants as a cause of neonatal diabetes and pancreatic agenesis, a case study describing a single family with neonatal diabetes caused by a homozygous *ZNF808* variant and 2 neonatal diabetes cohort studies which describe single families with *ZNF808* variants as part of a wider investigation of the genetic aetiologies present in a cohort of individuals with neonatal diabetes. All together, these publications describe 19 individuals, all with insulin-treated permanent neonatal diabetes and/or pancreatic agenesis. None of these studies investigated the presence of *ZNF808* variants in individuals with diabetes diagnosed after 6 months of age.

**Added value of this study**

This study investigates *ZNF808* variants in a large cohort of individuals with monogenic diabetes diagnosed between birth and 60 years of age. We report 17 additional individuals with *ZNF808*-diabetes, almost doubling the number of patients that have been described to date. We identified multiple individuals with transient diabetes and diabetes diagnosed after the neonatal period (range 6 months–23 years), broadening the phenotypic spectrum associated with this monogenic diabetes subtype. We also report an association between the position of variants within the *ZNF808* protein and diabetes phenotype, indicating that variant-specific differences in protein function may account for some of the phenotypic variability seen in the cohort.

**Implications of all the available evidence**

This study provides insights into the *ZNF808* monogenic diabetes subtype, showing that pathogenic *ZNF808* variants result in a much broader spectrum of diabetes phenotypes than first thought. Our results suggest that testing for variants in the *ZNF808* gene should be considered in individuals with diabetes diagnosed after the neonatal period, especially if born to related parents.

## Introduction

Loss-of-function variants in *ZNF808* have recently been identified as a cause of pancreatic agenesis (PA), clinically defined as neonatal diabetes (NDM) diagnosed in the first 6 months of life and exocrine pancreatic insufficiency.^1^ The identification of 15 individuals from 13 families with biallelic *ZNF808* variants and permanent neonatal diabetes conclusively established causality, as per the guidelines set out by *MacArthur* et al.^2^ All the individuals with biallelic *ZNF808* variants described by De Franco et al.^1^ had low birthweight and early onset of insulin-dependent diabetes, consistent with severe insulin deficiency pre- and postnatally.^3^

This discovery took the total number of disease genes for PA to eight, with dominantly-acting disease-causing variants reported in three genes (haploinsufficiency of *GATA6* and *GATA4,* and a specific missense variant in *CNOT1*)^4^, ^5^, ^6^ and recessive inheritance reported in 5 (loss-of-function variants in *PTF1A* gene body and enhancer, *PDX1*, *RFX6*, *ONECUT1* and *ZNF808*).^7^, ^8^, ^9^, ^10^, ^11^, ^12^ All these genes encode proteins involved in gene regulation, with 7 of these being transcription factors and 1 (CNOT1) being a transcriptional regulator. All 8 genes are expressed early during human development, although the exact developmental stage at which their expression peaks does vary between them, with some, for example *PDX1*, also having important roles in mature beta-cells.^13^ *ZNF808* expression peaks at the definitive endoderm and primitive gut tube stages, similar to *GATA4, GATA6* and *CNOT1* (Supplementary Figure S1).

Variability in the pancreatic phenotype has been reported for 4 genetic causes of PA (*GATA4*, *GATA6*, *PDX1* and *PTF1A* enhancer variants), either as the result of hypomorphic alleles or stochastic variation in the proliferation and differentiation of pancreatic cells.^4^^,^^5^^,^^14^^,^^15^ The observed differences in phenotype include variation in age at diabetes diagnosis, with onset in adolescence and adulthood reported in some patients; insulin-dependence, with some cases having transient forms of diabetes not requiring treatment and/or treated with oral hypoglycemic agents; and the presence and clinical manifestation of exocrine pancreatic insufficiency. Understanding the variability in the disease phenotype caused by variants in these genes is important as it can give insights into their biological function and inform genetic counselling for individuals with these rare diabetes subtypes.

In this study, we explore the phenotypic spectrum caused by biallelic *ZNF808* variants in a large cohort of individuals referred for monogenic diabetes testing.

## Methods

### Cohort

We screened for *ZNF808* variants in 4699 individuals referred to the Exeter Genomics Laboratory for monogenic diabetes genetic testing. In all individuals, comprehensive screening of the known monogenic diabetes genes had been performed but no disease-causing variants were identified. Of the 4699 individuals, 335 had neonatal diabetes (NDM) (diagnosis <6 months), 194 had infancy-onset diabetes (diagnosis between 6 and 12 months) and 4170 were referred with diabetes diagnosed after 1 year which was clinically suspected to have a monogenic aetiology (age at diagnosis range 12 months–60 years). In 69 individuals the diabetes had been reported as transient. Twelve individuals had clinically or biochemically confirmed pancreatic exocrine insufficiency. 232 of the individuals with diabetes diagnosed before one year were included in the previous *ZNF808* study.^1^ One individual from the previous *ZNF808* study (Patient 10 in De Franco et al.^1^) was not included in the cohort as genetic testing was not performed at the Exeter Genomics Laboratory. Clinical information and demographic information (age, sex) was provided by referring clinicians using standardised monogenic diabetes referral forms (available at www.diabetesgenes.org) and through clinical notes. As this was a study of an ultra-rare autosomal recessive disorder, sex was not considered during study design or participant recruitment.

### Genetic analysis

Genome sequencing was performed on leucocyte DNA from 549 individuals, for the remaining 4150 individuals the coding regions and intron/exon boundaries of the *ZNF808* gene were screened using targeted next-generation sequencing. Genomic data was processed using Genome Analysis Toolkit (GATK) best practice pipelines,^16^ with additional processing using the SavvySuite.^17^ This analysis allowed for the detection of single nucleotide variants, indels and large deletions/duplications. The data was processed as previously described^18^ and was interrogated to identify biallelic (either homozygous or compound heterozygous) coding variants affecting the *ZNF808* gene with a GnomAD v4^19^ minor allele frequency <0.0001. The identified variants were then classified using ACMG/ACGS guidelines, with only likely pathogenic and pathogenic variants retained (Supplementary Table S1).^20^, ^21^, ^22^ Genetic similarity of individuals to GnomAD^19^ ancestry groups was calculated using our own pipeline.^23^ Homozygosity mapping was performed using the SavvySuite^17^ to determine consanguineous parentage where it was not declared at referral.

### Ethics

The study was conducted in accordance with the Declaration of Helsinki, and all participants or their parents gave informed consent for genetic testing with ethical approval received from the Genetic Βeta-cell Research Bank, Exeter, U.K, which itself received ethical approval from the North Wales Research Ethics Committee, UK (IRAS project ID 231760).

### Statistics

A two-tailed Fisher's exact test was used to determine if there was a significant difference in the phenotypes associated with variants in the first and second half of the *ZNF808* protein. This test was appropriate as the data was categorical, independent and the marginal totals were fixed. The test was performed in R using base language functions.^24^ Inclusion in the study cohort was based upon detection of biallelic pathogenic/likely pathogenic *ZNF808* variants. Because this was a study of individuals with ultra-rare genetic disease, sample size determination, randomisation and blinding were not applicable.

### Role of funders

Funders had no role in the study design, data collection, data analyses, interpretation, or in the writing of the report.

## Results

**Fig. 1:**
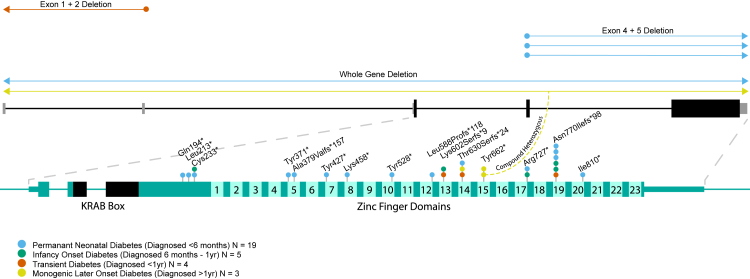
**Biallelic *ZNF808* variants identified in the Exeter monogenic diabetes cohort.** The variants are coloured by the phenotype of the individual they were found in.

We identified biallelic pathogenic/likely pathogenic *ZNF808* variants in 17 individuals from 15 families. This led to a total cohort of 31 individuals from 27 families after including the 14 individuals previously reported in De Franco et al.^1^ (clinical features summarised in Table 1, full data in Supplementary Table S2). Two of these newly identified individuals (4 and 8) have been included in separate cohort studies of neonatal diabetes in Sudan and Iraq.^25^^,^^26^ A total of 21 different *ZNF808* variants were identified in the cohort, 10 of which had not been previously reported. All of the *ZNF808* variants identified were either protein-truncating variants in the final exon of the gene (n = 15) or multi-exon deletions (n = 6) (Fig. 1). Thirty individuals, all born to related parents, were homozygous for *ZNF808* loss-of-function (LoF) variants; 1 individual (born to non-related parents) was compound heterozygous. No likely deleterious missense variants were identified.

**Table 1:** Clinical feature summary of individuals with Biallelic *ZNF808* variants.

	N individuals	Median age at diagnosis in weeks (IQR)	Median birth weight in sd from population median (IQR)	Current treatment	N with pancreatic exocrine insufficiency
PNDM	19	12 (4–20)	−2.58 (−3.07 to −2.27)	17 Insulin 1 Sulphonylurea 1 Unknown	5 Biochemically confirmed 5 Likely, based on clinical phenotype
Infancy-onset diabetes	5	30 (30–32)	−2.26 (−2.96 to −1.43)	5 Insulin	2 Likely, based on clinical phenotype
Transient diabetes	4	34 (17.07–34.5)	−2.32 (−2.69 to −1.6)	2 Insulin 2 No treatment (in remission)	1 Biochemically confirmed
Adolescent-onset diabetes	3	728 (624–962)	−1.34 (NA)^a^	2 Sulphonylurea 1 Insulin	None confirmed/likely
Overall	31	20 (6.5–30)	−2.48 (−3.01 to −2.11)	25 Insulin 3 Sulphonylurea 2 No treatment (in remission) 1 Unknown	6 Biochemically confirmed 7 Likely based on clinical phenotype

a Birth weight data unavailable for 2/3 individuals.

### Biallelic *ZNF808* pathogenic variants can cause diabetes with onset after the neonatal period

Seven newly identified individuals with biallelic *ZNF808* LoF variants had NDM. Including the 13 previously reported cases in the Exeter cohort, this brings the total number of individuals with NDM caused by *ZNF808* variants to 20, confirming that NDM is the most common diabetes phenotype associated with this genetic subtype (20/31, 64.5%).

In the 11 other individuals with biallelic *ZNF808* LoF variants, 10 had a confirmed diabetes onset after 6 months: 7 had infancy-onset diabetes between 6 and 12 months of age and 3 had diabetes diagnosed in adolescence^27^ (diagnosis at 10, 14 and 23 years). One individual did not have an exact age at diagnosis and was just known to have been diagnosed before 1 year.

### Biallelic *ZNF808* pathogenic variants can cause transient diabetes

In 4 newly identified individuals with biallelic *ZNF808* LoF variants the diabetes was reported to be transient: 1 individual had NDM (patient 11), 2 had infancy-onset diabetes (patients 9c, 12) and 1 had unspecified diabetes onset before 1 year (patient 10). All 4 individuals with transient diabetes had remitted by the age of 3 years, discontinuing insulin treatment. Subsequent relapse of diabetes and restart of insulin treatment was reported in 2 of the patients at the ages of 5 and 6.5 years.

### Variants present in individuals with non-PNDM phenotypes cluster in the latter half of the protein

10/12 individuals with diabetes phenotypes other than PNDM had truncating variants in the latter half of the protein (zinc finger 13 and beyond) (Fig. 1). The enrichment of variants in this region among individuals with non-PNDM phenotypes was statistically significant (two-tailed Fisher's exact p = 0.0143). Of the 6 variants within this region, 5 were found in at least one individual with a non-PNDM phenotype, with 2/6 being found exclusively in these individuals. The remaining 2 individuals with non-PNDM phenotypes had a deletion which included the *ZNF808* promoter region and the first 2 non-coding exons of the gene and a p.(Cys233∗) stop gain variant.

### Diabetes caused by biallelic *ZNF808* pathogenic variants does not always require insulin treatment

Three individuals with biallelic *ZNF808* LoF variants were treated with sulphonylureas at last contact: one (patient 1) was diagnosed with PNDM at 13 weeks, whilst the other two (patients 10 and 11) had adolescent-onset diabetes at 23 and 14 years, respectively. Both individuals with adolescent-onset diabetes who were sulphonylurea-treated were reported to have persistent stable glycaemia without insulin treatment, indicating that the sulphonylurea treatment was effective. The individual with PNDM who was sulphonylurea-treated was 10 months of age at last contact and thus stable glycaemia in response to the treatment could not be confirmed. Twenty-five of the remaining 28 individuals were insulin-treated at the last assessment. The median insulin dose was 0.8 U/kg/day (IQR = 0.5 U/kg/day–2 U/kg/day, data available for 11 individuals). Two further individuals were in diabetes remission and not receiving insulin treatment. Treatment details were not available for one individual.

### Birth weight is variable in individuals with biallelic *ZNF808* disease-causing variants

The median birthweight in the cohort was −2.48SD (IQR = −3.01SD to −2.11SD, data available for 24 individuals). 19/24 individuals were born severely small for gestational age (defined as <3rd centile or 1.88SD below the median). 2/24 were born small for gestational age (defined as <10th centile or 1.28SD below the median). The remaining 3 individuals were born at a weight appropriate for gestational age (defined as >10th centile and <90th centile or within 1.28SD of the median).

The 5 individuals who did not have severe IUGR showed either diabetes onset after the neonatal period or did not consistently require insulin treatment: 1 individual (patient 13) had adolescent-onset diabetes (−1.34SD), 1 (patient 11) had transient diabetes (0.14 SD), 2 individuals (7, 8) had infancy-onset diabetes (−0.9SD and −1.61SD) and 1 (patient 1) had PNDM diagnosed at 13 weeks of age which did not require insulin treatment (treated with sulphonylureas, last contact at 10 months of age) (−0.82SD).

### Biallelic *ZNF808* variants are a cause of diabetes without pancreatic exocrine insufficiency

Biochemical assessment of pancreatic exocrine function through fecal elastase measurement was performed in 7/31 individuals. One (patient 13), diagnosed with diabetes at 14 years, had a normal fecal elastase level (>200 μg/g stool), excluding pancreatic exocrine insufficiency (PEI). The remaining 6 had biochemically confirmed PEI, consistent with a clinical diagnosis of pancreatic agenesis/hypoplasia. Seven of the remaining 24 individuals for whom fecal elastase testing was not available had clinical features of PEI, such as failure to thrive, steatorrhoea and low iron, folate or vitamin D. No clinical features suggestive of PEI were reported in the other 17.

Pancreatic imaging results were available for 2 individuals with biochemically confirmed PEI (one newly identified; patient 10). In both individuals only the pancreas head was visualised. PEI was not isolated to individuals with PNDM; individual 12, who had biochemically confirmed PEI, had transient diabetes that was diagnosed at birth and was not insulin treated between the ages of 3 months and 5 years, despite requiring enzyme replacement therapy for PEI during this same period.

## Discussion

Our study in a large monogenic diabetes cohort expands the phenotype resulting from *ZNF808* LoF variants from PNDM and PA to infancy-onset, transient and adolescent-onset diabetes without exocrine pancreatic insufficiency. The majority of individuals with *ZNF808* loss-of-function variants (19/31) had PNDM, however 12 individuals had other diabetes phenotypes: 5 infancy-onset diabetes, 4 transient diabetes and 3 adolescent-onset diabetes. The identification of biallelic variants among individuals with adolescent-onset diabetes has implications for genetic testing and indicates that *ZNF808* testing should be considered in individuals with maturity onset diabetes of the young (MODY).

Our results show that loss of *ZNF808* can result in a milder pancreatic phenotype, that does not always require insulin treatment and can include normal pancreatic exocrine function. This is exemplified by patient 13 who was diagnosed with diabetes at 14 years, is treated with a sulphonylurea and was biochemically confirmed to have normal pancreatic exocrine function. In total, 7 individuals in our cohort exhibited prolonged periods without insulin treatment: 3 are currently treated with sulphonylurea and 4 had transient diabetes. Two of the sulphonylurea-treated individuals had adolescent-onset diabetes and were reported to have persistent stable glycaemia while on treatment, supporting its efficacy. However, the current efficacy does not guarantee that insulin treatment will not be required for these two individuals in the future. A greater degree of endogenous insulin secretion in the non-insulin-treated individuals is corroborated by their higher birth weights compared to the rest of the cohort. Insulin is a key driver of foetal growth in the final trimester of pregnancy and as such the elevated birth weights suggest a greater degree of in utero insulin secretion.^3^ The effect of loss of *ZNF808* on the exocrine pancreas also appears to be variable; 17 individuals had no reported symptoms of exocrine insufficiency, in addition to patient 13 who had normal faecal elastase. The variation in exocrine and endocrine features among individuals with biallelic *ZNF808* LoF variants suggests a variable effect of *ZNF808* dysfunction on the differentiation of cells into the pancreatic lineage during foetal development.

Our results are consistent with the hypothesis that, in some individuals with biallelic *ZNF808* variants, sufficient pancreatic cells are produced for proper exocrine function and that the number of beta cells produced can be enough to delay the onset of diabetes or allow for extended periods of time without insulin treatment. However, homozygous pathogenic and likely pathogenic *ZNF808* variants are absent from healthy individuals in the GnomAD, UK Biobank, Genes and Health, and All of Us population cohorts,^19^^,^^28^, ^29^, ^30^ suggesting that the variants are fully penetrant but show variable expressivity. Disease-causing *ZNF808* variants were also not identified in individuals with diabetes in these population cohorts. This is not unexpected since these cohorts include only a small number of individuals with monogenic diabetes and biallelic *ZNF808* variants were rare in our clinically selected cohort of individuals with adolescent/adult-onset monogenic diabetes (3/4170 individuals diagnosed after 1 year of age).

The significant clustering of *ZNF808* variants in individuals with transient, infancy-onset and adolescent-onset diabetes in the latter half of the protein suggests that truncation in this region may lead to a greater degree of retained function, with a more variable effect on pancreas development. However, some of the variants in these regions were also identified in individuals with PNDM and PA, meaning a genotype-phenotype association does not alone explain the greater preservation of pancreatic function observed in some patients. The reason for the variable expressivity observed in the variants in this region is unclear. Whilst genotype-phenotype correlations have been suggested to explain the variable penetrance and expressivity of the pancreatic phenotype in some PA subtypes, for example, hypomorphic variants in *PDX1*^15^ and a non-coding modifier in *GATA6*,^31^ the variability of the pancreatic phenotype associated with most PA genes is still unexplained. The biallelic *ZNF808* genotype of individuals with adolescent-onset diabetes also distinguishes the disease caused by *ZNF808* variants from other PA genes that cause both PNDM and adolescent/adult-onset diabetes, such as *RFX6* and *PDX1*, where the later-onset diabetes occurs through dominantly acting variants, while neonatal-onset diabetes is caused by recessively acting variants.^7^^,^^8^^,^^15^^,^^32^ Further studies are needed to fully establish the mechanisms underlying the variable phenotype in individuals with *ZNF808* variants.

There were some limitations to this study. We were unable to organise faecal elastase testing in 24 individuals; many participants were from remote regions without access to local biochemical labs where the testing could be performed. This means that we are reliant on clinical reports of features that are consistent with exocrine insufficiency, which are not as reliable for establishing exocrine function as biochemical testing. This meant that we could not fully evaluate correlation between pancreatic endocrine and exocrine function or investigate any genotype-phenotype association between *ZNF808* variants and exocrine function. Additionally, our monogenic diabetes cohort is enriched for individuals diagnosed with diabetes before 6 months of age. Children and adolescents diagnosed after this age have a much higher chance of type 1 diabetes, so are significantly less likely to undergo genetic testing for monogenic diabetes. This means that the relative frequencies of the different diabetes phenotypes observed in this study are not necessarily representative of the true distribution among individuals with bi-allelic *ZNF808* variants.

In summary, we expand the phenotypic spectrum associated with biallelic LoF variants in *ZNF808*, which ranges from PA with complete ablation of exocrine and endocrine function to transient and adolescent-onset diabetes with normal exocrine function. The identification of biallelic variants among individuals diagnosed with diabetes in adolescence and individuals with non-insulin-treated diabetes has implications for treatment and genetic testing and indicates that inclusion of *ZNF808* to genetic testing panels used for MODY should be considered.

## Contributors

J.R.S., E.D.F. and A.T.H. participated in study conception and design. E.D.F., S.E.F., A.T.H., K.C., S.A.A. and K.A.P. recruited patients. The ZNF808 clinical consortium performed blood draws and provided clinical information. J.R.S., E.D.F, M.B.J., K.C., K.A.P and M.N.W. analysed the sequencing data. J.R.S. carried out statistical analysis. J.R.S. wrote the first draft of the manuscript. E.D.F., M.B.J., N.D.L.O., A.T.H., S.E.F. and the Human-Specific Pancreatic Development Consortium participated in manuscript improvement. All authors reviewed the manuscript. E.D.F. and J.R.S. had full access to all the data in the study and take responsibility for the integrity of the data and the accuracy of the data analysis. All authors read and approved the final version of the manuscript.

## Data sharing statement

Clinical and genotype data can be used to identify individuals and is therefore available through collaboration to experienced teams working on approved studies examining the mechanisms, cause, diagnosis and treatment of diabetes and other beta-cell disorders. Requests for collaboration will be considered by a steering committee following an application to the Genetic Beta Cell Research Bank (https://www.diabetesgenes.org/current-research/genetic-beta-cell-research-bank/). Contact by email should be directed to Prof Elisa De Franco (e.de-franco@exeter.ac.uk). All requests for access to data will be responded to within 14 days.

## Declaration of interests

The authors confirm there are no relevant conflicts of interest pertinent to this work. The Human-Specific Pancreatic Development Consortium was funded by a Wellcome Trust Collaborative Award in Science (224600/Z/21/Z). The *ZNF808* clinical consortium did not receive any external funding.

## Appendix A Supplementary data

Supplementary Figure and Tables

Consortium member names for pubmed
