# Supplementary material for: Loss of function variants in the primate-specific gene ZNF808 cause neonatal, transient and adult-onset diabetes
Source: eBioMedicine. 2026 Jan 6;124:106113. doi: 10.1016/j.ebiom.2025.106113 (PMC12808904; doi:10.1016/j.ebiom.2025.106113)
Supplement: Supplementary Figure and Tables [file mmc1.docx]

**Loss of function variants in the primate-specific gene *ZNF808* cause neonatal, transient and adult-onset diabetes - supplementary data**

**Table of Contents**

Members of the *ZNF808* Clinical Consortium – Page 2

Members of the Human-Specific Pancreatic Development Consortium – Page 2

Supplementary table 1: ACMG/ACGS classification of newly identified bi-allelic *ZNF808* variants in the Exeter monogenic diabetes cohort. – Page 3

Supplementary Table 2: Clinical data of newly identified patients with bi-allelic *ZNF808* variants in the Exeter cohort. – Page 4

Supplementary Figure 1: Expression of known pancreatic agenesis associated genes across development of embryonic stem cell-derived pancreatic islets. – Page 6

**Members of the *ZNF808* Clinical Consortium:**

Mohamed A Abdullah^1^, Hessa Alkandari^2,3^, Zehra Aycan^4^, Semra Çetinkaya^4^, Nancy Elbarbary^5^, Radha Ghildiyal^6^, Susana Gonzalez^7^, Shaun Gorman^8^, Samar Hassan^9^, Savita Khadse^6^, Jan Lebl^10^, Jaida Manzoor^11^, Nikhil Shah^6^, Tara Hussein Tayeb^12^

1. Department of Paediatrics and Child Health, Faculty of Medicine, University of Khartoum, Sudan.
2. Department of Population Health, Dasman Diabetes Institute, Kuwait
3. Department of Pediatrics, Farwaniya Hospital, Kuwait
4. Clinic of Pediatric Endocrinology, Dr. Sami Ulus Children's Training and Research Hospital, Ankara, Turkey.
5. Diabetes and Endocrine Unit, Department of Pediatrics, Faculty of Medicine, Ain Shams University, Cairo, Egypt.
6. Department of Pediatrics, Lokmanya Tilak Municipal Medical College and Lokmanya Tilak Municipal General Hospital, Mumbai, India.
7. Bradford Royal Infirmary, Bradford, United Kingdom.
8. Bradford Teaching Hospitals NHS Foundation Trust, Bradford, UK.
9. Department of Pediatric Endocrinology, Gaafar Ibn Auf Pediatric Tertiary Hospital, Khartoum, Sudan.
10. Department of Pediatrics, 2nd Faculty of Medicine, Charles University and Motol University Hospital, Prague, Czechia
11. The Children's Hospital University of Child Health Sciences, Lahore, Pakistan.
12. Department of Paediatrics, Sulaimani University, College of Medicine, Sulaimani, Iraq.

**Members of the Human-Specific Pancreatic Development Consortium:**

Alaa Al Assi^1^, Arya Anil^1^, Diego Balboa^2^, Urvashi Chitnavis^3^, Juliette Davies^3^, Doga Eskier^1^, Michael Imbeault^4^, Santiago Morell^3^, Sachin Muralidharan^2^, Timo Otonkoski^2^, Jonna Saarimäki-Vire^2^

1. Department of Clinical and Biomedical Sciences, University of Exeter, Exeter, United Kingdom
2. Stem Cells and Metabolism Research Program, Faculty of Medicine, University of Helsinki, Helsinki, Finland.
3. Department of Genetics, University of Cambridge, Cambridge, UK.
4. Department of Medical & Molecular Genetics, King’s College London, London, UK

| **Variant** | **Classification** | **Score** | **Criteria** |
| --- | --- | --- | --- |
| SNVs/Indels | | | |
| NM_001039886.4:c.1113C>A,  NP_001034975.2:p.(Tyr371*) | Likely Pathogenic | 7 | PVS1_strong: Last exon variant removes >10% of amino acids  PM2: AF of 1.24e-6 in GnomAD v4 – no homozygotes  PM3_supporting: homozygous (1 in cohort) |
| NM_001039886.4:c.1805_1806del,  NP_001034975.2:p.(Lys602Serfs*9) | Likely Pathogenic | 8 | PVS1_strong: Last exon variant removes >10% of amino acids  PM2: AF of 6.20e-6 in GnomAD v4 – no homozygotes  PM3_moderate : homozygous (2+ in cohort) |
| NM_001039886.4:c.1889_1890del, NP_001034975.2:p.(Thr630Serfs*24) | Likely Pathogenic | 8 | PVS1_strong: Last exon variant removes >10% of amino acids  PM2: AF of 6.82e-6 in GnomAD v4 – no homozygotes  PM3_moderate: homozygous (2+ in cohort) |
| NM_001039886.4:c.1985dup,  NP_001034975.2:p.(Tyr662*) | Likely Pathogenic | 8 | PVS1_strong: Last exon variant removes >10% of amino acids  PM2: AF of 1.73e-5 in GnomAD v4 – no homozygotes  PM3_ moderate: In trans with pathogenic deletion |
| NM_001039886.4c.2179A>T,  NP_001034975.2:p.(Arg727*) | Likely Pathogenic | 8 | PVS1_strong: Last exon variant removes >10% of amino acids  PM2: Absent from GnomAD v4  PM3_moderate: homozygous (2+ in cohort) |
| NM_001039886.4:c.2309del,  NP_001034975.2:p.(Asn770Ilefs*98) | Likely Pathogenic | 8 | PVS1_strong: Last exon variant removes >10% of amino acids  PM2: Absent from GnomAD v4  PM3_moderate: homozygous (2+ in cohort) |
| NM_001039886.4:c.2428_2429del,  NP_001034975.2:p.(Ile810*) | Likely Pathogenic | 7 | PVS1_strong: Last exon variant removes >10% of amino acids  PM2: AF of 4.96e-6 in GnomAD v4 – no homozygotes  PM3_supporting: homozygous (1 in cohort) |
| Large Deletions | | | |
| NC_000019.9:g.52975809_53044081del | Likely Pathogenic | 0.90 | CNV-1A: deletion contains functionally important regulatory element (entirety of promoter) |
| NC_000019.9:g.53056673_53057794_53107506_53116060del | Pathogenic | 1.00 | CNV-2C-1: Significant portion of coding gene deleted |
| NC_000019.9:g.53048579_53066289del | Pathogenic | 1.00 | CNV-2C-1: Significant portion of coding gene deleted |
| NC_000019.9:g.53016756_53061297del | Pathogenic | 1.00 | CNV-2A: Entirety of gene deleted |

**Supplementary table 1: ACMG/ACGS classification of newly identified bi-allelic *ZNF808* variants in the Exeter monogenic diabetes cohort.** Single Nucleotide Variants = SNV, CNV = Copy Number Variants. SNV/Indels points thresholds: 6-9 Likely Pathogenic, ≥10 Pathogenic and CNV points thresholds: 0.90-0.98 = Likely Pathogenic, ≥0.99 = Pathogenic.^19-21^ Variants described using HGVS nomenclature based on RefSeq NM_001039886.4.

**Supplementary Table 2: Clinical data of newly identified patients with bi-allelic *ZNF808* variants in the Exeter cohort.** Genetic ancestry group classifications are based on genetic similarity to reference groups: MID – Middle Eastern. OTH – other. EAS – East Asian. AFR – African. SAS – South East Asian.

| **Patient** | **Age at Last Contact** | **Sex** | **Genetic Ancestry**  **Group** | **Parents Related** | ***ZNF808* variant** | **Diabetes phenotype** | **Age Diagnosed with Diabetes** | **Age Diabetes Remitted/**  **Relapsed** | **Birthweight** | **Initial Treatment (Dose)** | **Current Treatment (Dose)** | **C-peptide pmol/l** | **Pancreatic Exocrine Insufficiency** |
| --- | --- | --- | --- | --- | --- | --- | --- | --- | --- | --- | --- | --- | --- |
| 1 | 10 Months | F | SAS | Known to be 1st Cousins | p.(Thr630Serfs*24)/p.(Thr630Serfs*24) | PNDM | 13 Weeks | N/A | 2500g/  37 Weeks  (-0.82SD/20.6 centile) | Glibenclamide (1.25mg/day) | Glibenclamide (5mg/day) | Unknown | No known clinical signs of malabsorption |
| 2 | 5 Years | M | MID | Known to be 1st Cousins | p.(Ile810*)/ p.(Ile810*) | PNDM | 8 Weeks | N/A | 2000g/  38 Weeks  (-2.58SD/0.5 centile) | Insulin (1.3u/kg/day) | Insulin (0.38u/kg/day) | Unknown | No known clinical signs of malabsorption |
| 3 | 21 days | M | EAS | Found by homozygosity mapping | NC_000019.9:g.53048579_53066289del/NC_000019.9:g.53048579_53066289del | PNDM | 2 weeks | N/A | 1645g/  36 Weeks  (-2.5SD/0.6 centile) | Unknown | Insulin (0.22u/kg/day) | Unknown | No known clinical signs of malabsorption |
| 4 | 1.5 Years | M | AFR | Known to be 1st Cousins | p.(Asn770Ilefs*98)/ p.(Asn770Ilefs*98) | PNDM | 20 Weeks | N/A | 1800g/  Unknown | Insulin (0.5u/kg/day) | Insulin (1.3u/kg/day) | Unknown | No known clinical signs of malabsorption |
| 5 | 2 Years | F | MID | Found by homozygosity mapping | p.(Tyr371*)/ p.(Tyr371*) | PNDM | 12 Weeks | N/A | 2000g/  37 Weeks  (-2.16SD/1.5 centile) | Insulin (0.3u/kg/day) | Insulin (0.9u/kg/day) | 3.3 | No known clinical signs of malabsorption |
| 6 | 1.5 Months | F | SAS | Found by homozygosity mapping | NC_000019.9:g.53056673_53057794_53107506_53116060del/NC_000019.9:g.53056673_53057794_53107506_53116060del | PNDM | 6 Weeks | N/A | 1490g/  37 Weeks  (-3.3SD/0 centile) | Unknown | Insulin (1.3u/kg/day) | <30 | Likely (clinical signs of malabsorption) |
| 7 | 6 Years | M | OTH | Found by homozygosity mapping | p.(Lys602Serfs*9)/ p.(Lys602Serfs*9) | Infancy-onset Diabetes | 30 Weeks | N/A | 1300g/  31 Weeks  (-0.9SD/  18.4 centile) | Glibenclamide (2.5mg/day) + Occasional insulin (2u) | Insulin (0.5u/kg/day) | Unknown | Likely (clinical signs of malabsorption) |
| 8 | 13 Years | M | OTH | Known to be 2nd Cousins | p.(Arg727*)/ p.(Arg727*) | Infancy-onset Diabetes | 28 Weeks | N/A | 2600g/  39 Weeks  (-1.61SD/5.34 centile) | Insulin (Unknown dose) | Insulin (Unknown dose) | Unknown | Likely (pancreatic hypoplasia confirmed on ultrasound) |
| 9a | 4 Years | M | AFR | Known to be 1st Cousins | p.(Asn770Ilefs*98)/ p.(Asn770Ilefs*98) | Infancy-onset Diabetes | 32 Weeks | N/A | 2000g/  39 Weeks (-2.9SD/0.2 centile) | Insulin (Unknown dose) | Insulin (Unknown dose) | 56.61 | No known clinical signs of malabsorption |
| 9b | 10 Months | F | AFR | Known to be 1st Cousins | p.(Asn770Ilefs*98)/ p.(Asn770Ilefs*98) | Infancy-onset Diabetes | 30 Weeks | N/A | Unknown | Insulin (Unknown dose) | Insulin (Unknown dose) | Unknown | No known clinical signs of malabsorption |
| 9c | 6.5 Years | M | AFR | Known to be 1st Cousins | p.(Asn770Ilefs*98)/ p.(Asn770Ilefs*98) | Transient Diabetes | 34 Weeks | 1.5y/Not Yet | 2500g/  40 Weeks  (-2.18SD/1.4 centile) | Insulin (Unknown dose) | None | Unknown | No known clinical signs of malabsorption |
| 10 | 6.5 Years | M | OTH | Known to be 3rd Cousins | NC_000019.9:g.52975809_53044081del/ NC_000019.9:g.52975809_53044081del | Transient Diabetes | <1 year | 3y/6.5y | 2750g/  36 Weeks (0.14SD/  55.7 centile) | Unknown | Insulin (0.3u/kg/day) | 258 | No known clinical signs of malabsorption |
| 11 | 21 Years | F | SAS | Known to be 1st Cousins | p.(Thr630Serfs*24)/ p.(Thr630Serfs*24) | Transient Diabetes | 1 Day | 3m/5y | 1430g/  37 Weeks  (-3.42SD/0 centile) | Insulin (Unknown dose) | Insulin (0.9u/kg/day) | 170 | Yes (Biochemically Confirmed) |
| 12 | 2 Years | M | NFE | Found by homozygosity mapping | p.(Lys602Serfs*9)/ p.(Lys602Serfs*9) | Transient Diabetes | 35 Weeks | Unknown/Not Yet | 2230g/  39 Weeks  (-2.45SD/0.7 centile) | Unknown | None | 589 | No known clinical signs of malabsorption |
| 13 | 21 Years | M | NFE | No | p.(Tyr662*)/ NC_000019.9:g.53016756_53061297del | Adolescent-onset Diabetes | 14 Years | N/A | 3100g/  42 Weeks (-1.34SD/9 centile) | Insulin (Unknown dose) | Glimepiride (1mg) | Unknown | No (Biochemically Confirmed) |
| 14 | 28 Years | F | NFE | Found by homozygosity mapping | p.(Thr630Serfs*24)/ p.(Thr630Serfs*24) | Adolescent -onset Diabetes | 23 Years | N/A | Unknown | Diet | Glimepiride (1mg) + Metformin (2000mg) | Unknown | No known clinical signs of malabsorption |
| 15 | 34 Years | F | SAS | Found by homozygosity mapping | p.(Tyr662*)/ p.(Tyr662*) | Adolescent -onset Diabetes | 10 Years | N/A | Unknown | Insulin (Unknown dose) | Insulin (Unknown dose) | Unknown | No known clinical signs of malabsorption |


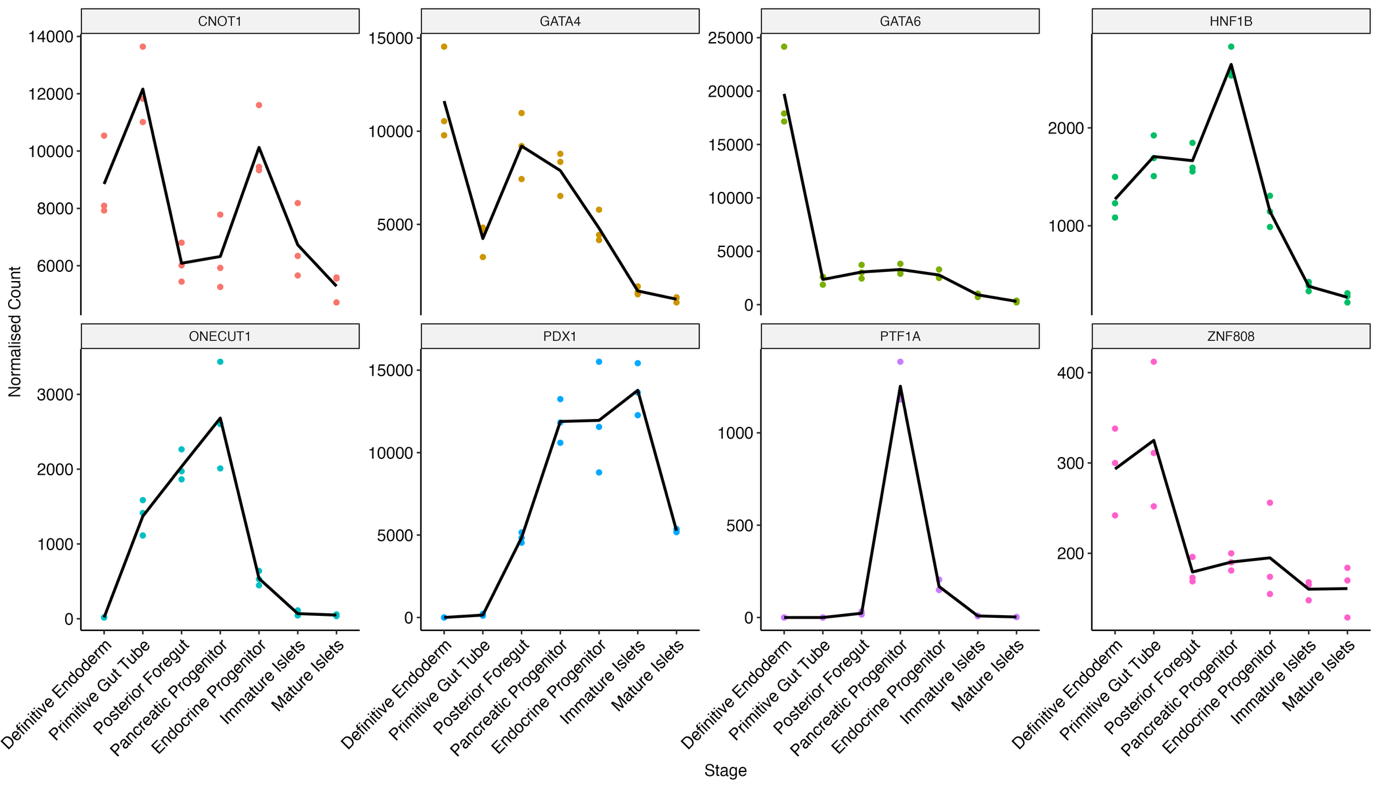


**Supplementary Figure 1: Expression of known pancreatic agenesis associated genes across development of embryonic stem cell-derived pancreatic islets.** Expression data comes from RNA sequencing performed on wild type cells in *De Franco et al.*^1^
