# Supplementary material for: Loss of function variants in the primate-specific gene ZNF808 cause neonatal, transient and adult-onset diabetes
Source: eBioMedicine. 2026 Jan 6;124:106113. doi: 10.1016/j.ebiom.2025.106113 (PMC12808904; doi:10.1016/j.ebiom.2025.106113)
Supplement: Consortium member names for pubmed [file mmc2.docx]

***ZNF808* clinical consortium members**

| **First names** | **Surnames** |
| --- | --- |
| Mohamed | Abdullah |
| Hessa | Alkandari |
| Zehra | Aycan |
| Semra | Çetinkaya |
| Nancy | Elbarbary |
| Radha | Ghildiyal |
| Susana | Gonzalez |
| Shaun | Gorman |
| Samar | Hassan |
| Savita | Khadse |
| Jan | Lebl |
| Jaida | Manzoor |
| Nikhil | Shah |
| Tara Hussein | Tayeb |

**Human-Specific Pancreatic Development Consortium members**

| **First names** | **Surnames** |
| --- | --- |
| Alaa | Al Assi |
| Arya | Anil |
| Diego | Balboa |
| Urvashi | Chitnavis |
| Juliette | Davis |
| Doga | Eskier |
| Michael | Imbeault |
| Santiago | Morell |
| Sachin | Muralidharan |
| Timo | Otonkoski |
| Jonna | Saarimäki-Vire |
|  |  |
|  |  |
|  |  |
|  |  |
|  |  |
|  |  |
|  |  |
|  |  |
|  |  |
|  |  |
|  |  |
|  |  |
|  |  |
|  |  |
